# Royal jelly causes hypotension and vasodilation induced by increasing nitric oxide production

**DOI:** 10.1002/fsn3.970

**Published:** 2019-02-17

**Authors:** Yongming Pan, Yili Rong, Mengmeng You, Quanxin Ma, Minli Chen, Fuliang Hu

**Affiliations:** ^1^ College of Animal Sciences Zhejiang University Hangzhou China; ^2^ Comparative Medical Research Institute, Experimental Animal Research Center Zhejiang Chinese Medical University Hangzhou China

**Keywords:** acetylcholine, calcium channels, nitric oxide, royal jelly, vasorelaxation

## Abstract

Among royal jelly’s (RJ) various biological activities, its possible antihypertension and vasorelaxation effects deserve particular attention, but the underlying mechanisms of action remain unclear. Therefore, this study used the spontaneously hypertensive rats (SHR) hypertension model and the isolated rabbit thoracic aorta rings model to explore the mechanisms underlying the hypotension and vasorelaxation effects of RJ. Rats were divided into the following groups (*n* = 6): WKY‐control group, SHR‐control group, and SHR‐RJ group. SHR‐RJ group was received 1 g/kg of RJ via oral administration daily for 4 weeks. Systolic blood pressure (SBP), diastolic blood pressure (DBP), heart rate (HR), and nitric oxide (NO) level were detected. In addition, the mechanism of vasodilation of RJ was investigated using an isolated rabbit aortic ring technique. RJ significantly reduced SBP and DBP as well as increased NO levels of SHR in vivo. RJ caused vasorelaxation of the isolated aorta rings, and this effect was inhibited by atropine (M_3_ receptor blocker), L‐NAME (nitric oxide synthase inhibitor), methylene blue (guanylate cyclase inhibitor), and indomethacin (cyclooxygenase inhibitor). Moreover, RJ could markedly suppress the NE‐induced intracellular Ca^2+^ releases and high K^+^‐induced extracellular Ca^2+^ influx in denuded aortic rings. In addition, RJ can also increase cGMP levels and the production of NO in isolated aortic rings. The present study showed that RJ has antihypertensive effects and was associated with increased NO production. In addition, RJ contains muscarinic receptor agonist, possibly an acetylcholine‐like substance, and induces vasodilation through NO/cGMP pathway and calcium channels.

## 1 INTRODUCTION

Hypertension is an increasingly important public health issue and a major risk factor for global disease burden. According to 2017 China Cardiovascular Disease Report, 270 million people in China suffered from hypertension (Chen et al., 2018). On the same year, the American College of Cardiology (ACC) and the American Heart Association (AHA) introduced new guidelines to update the definition of hypertension, which lowered the standards of hypertension to 130/80 mmHg instead of 140/90 mmHg (Whelton et al., 2018), further to stress the importance of early detection and intervention of hypertension. With the improvement in human health management awareness, functional foods with medicinal value have lowered the risk of illness of late years. Royal Jelly (RJ), secreted by the hypopharyngeal and mandibular glands of worker honeybees (Isidorov, Czyzewska, Isidorova, & Bakier, 2009), has served as a health functional food for more than two centuries. The composition of RJ contains water (50%–60%), proteins (18%), carbohydrates (15%), lipids (3%–6%), minerals (1.5%), and vitamins (Nagai & Inoue, 2004). In addition, RJ was reported to contain other bioactive ingredients such as proteins, fatty acids, adenosine, estradiol, and acetylcholine. (Khalil, 2006). RJ exerts a wide range of biological effects on promoting human health, such as antioxidation, anti‐inflammatory, and antihypertension effects (Feng et al., 2015)， which may lead to preventing cardiovascular and cerebrovascular diseases.

Impairment of vascular endothelium is one of the pathophysiological conditions of hypertension, diabetes, atherosclerosis, and other diseases, which is linked to the decrease in nitric oxide (NO) and the increase in oxygen free radicals (Martinez‐Revelles et al., 2013; Sandoo, van Zanten, Metsios, Carroll, & Kitas, 2010). Restoring endothelial function or complement NO treatment is important to preventing hypertension. Thus, some studies have been conducted on the active component of RJ for lowering blood pressure. Fatty acids in RJ mostly exist in free forms, such as trans‐10‐hydroxy‐2‐decenoic acid (10‐H2DA), 10‐hydroxydecanoic acid (10‐HDA), and sebacic acid (Li, Huang, & Xue, 2013; Terada, Narukawa, & Watanabe, 2011). Among them, 10‐H2DA is considered to have vasodilation activity and antihypertensive ability (Okuda, Kameda, & Morimoto, 1998). The major royal jelly proteins (MRJPs) are also essential proteins in RJ, such as MRJP1 with antihypertensive activity (Fan et al., 2016). Besides, it is also noted that RJ contains rennin and angiotensin I‐converting enzyme (ACE) dual inhibitory peptides, which can lower blood pressure in spontaneously hypertensive rats (SHR) (Sultana et al., 2008; Takakidoi, Hashimoto, Yamamura, & Kamei, 2009; Tokunaga et al., 2004). Moreover, RJ causes temporary vasodilation on dog’s femoral arteries (Shinoda et al., 1978). Nevertheless, the mechanism of RJ’s hypotensive and vasorelaxation effects remains unclear. Therefore, we hypothesized that RJ’s hypotensive and vasodilative mechanisms may be related to the increased NO production. In order to test this hypothesis, we studied that whether RJ can cause hypotension and vasodilation, and how does RJ exert vasodilation activity, and whether this activity is associated with the increased NO production.

## 2 MATERIALS AND METHODS

### 2.1 Drug and reagents

**Figure 1:**
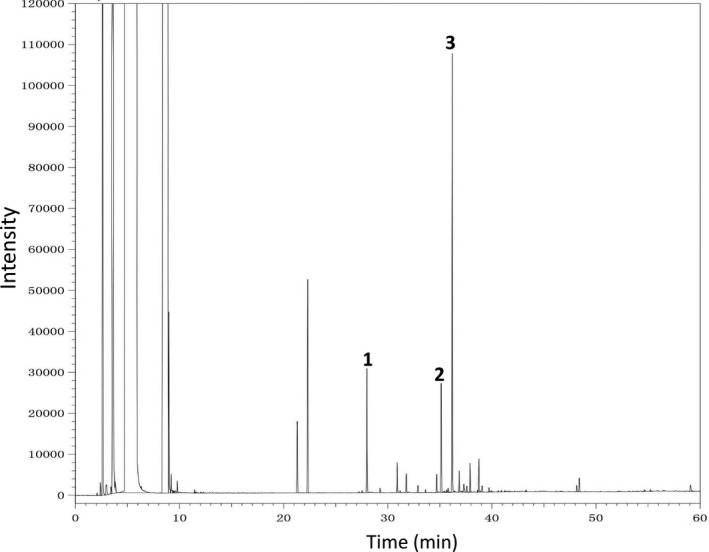
The gas chromatogram of royal jelly’s (RJ). 1. Methyl paraben (MeP, internal standard); 2. 10‐hydroxydecanoic acids (10‐HDA); 3. 10‐hydroxy‐2‐decenoic acid (10‐H2DA)

Fresh RJ was obtained from College of Animal Science, Zhejiang University (Hangzhou, China), during 2016 and kept on −20℃ until use. RJ was standardized with the amounts of specific fatty acid 10‐H2DA and 10‐HDA. RJ contained a minimum 1.91% of 10‐H2DA and a minimum 0.54% of 10‐HDA (Figure 1). RJ was dissolved in normal saline and given orally. Norepinephrine (NE), acetylcholine (ACh), L‐NAME, and atropine were purchased from Sigma‐Aldrich (St Louis, MO, USA). Methylene blue (MB) was obtained from Shanghai Science Reagent Co. LTD (China). NO and cGMP kits were purchased from Nanjing Institute of Bioengineering (China). Other reagents were of analytical reagent.

### 2.2 Animals

Male spontaneously hypertensive rats (SHR) and Wistar Kyoto rats (WKY), at 8 weeks of age were obtained from SLAC Laboratory Animal Co., Ltd. (Shanghai, China). Japanese White Rabbits (male, 4 months old) were supplied by Experimental Animal Research Center, Zhejiang Chinese Medical University (Zhejiang, China). Rats and rabbits were respectively housed under controlled temperature (21 ± 1°C) and lighting cycle (light on 07:00–19:00). In addition, all animals had free access to water and food. All experimental protocols and procedures were reviewed and approved by the Institutional Animal Care and Use Committee of the Zhejiang Chinese Medical University (Protocol ZSLL‐2016‐115).

### 2.3 In vivo antihypertensive study of RJ

After 2 weeks of prefeeding, the rats were divided into the following groups (*n* = 6 per group): WKY‐control group, SHR‐control group, and SHR‐RJ group. SHR‐RJ group was received 1 g/kg of RJ via oral administration daily for 4 weeks. Systolic blood pressure (SBP), diastolic blood pressure (DBP), and heart rate (HR) were monitored by tail‐cuff methods (ALC‐NIBP, Alcott Biotech Co, Ltd, Shanghai, China) every week. The rats were trained for at least 3 days prior to the blood pressure measurements in this study. After 4 weeks of treatment, all rats fasted for 8 hr, and then 0.6 ml blood samples were drawn from their tail vein with heparin anticoagulation, from which blood plasma was separated. The activities of nitro oxidase (NO) in the serum were measured according to the procedures of colorimetric commercial kit (Jiancheng Bioengineering Institute, Nanjing, China).

### 2.4 In vitro vasorelaxation studies of RJ

The method of preparation of rabbit aortic rings has been described previously (Shou et al., 2012). Isolated rabbit aortic rings were suspended in organ chambers containing 5 ml Krebs–Henseleit (K‐H) solution, placed between 2 stainless steel hooks, and connected to an isometric force transducer. Changes in tension were recorded by a MedLab Biological Signal Collection System (MedEase Science and Technology, Nanjing, China). In some aorta rings, the endothelium was denuded gently by rubbing the inner surface with a wet cotton ball. After that, the intactness and function of endothelium were tested. If ACh (10 μM) could induce more than 80% relaxation in NE (1 μM)‐contracted aorta rings, then the endothelium is intact. However, aorta rings would be considered completely denuded endothelium when Ach could just induce < 10% relaxation in NE‐contracted aorta rings.

#### 2.4.1 Effects of RJ on NE‐induced contraction

Endothelium‐intact or endothelium‐denuded rings were precontracted with NE (1 μM) in standard K‐H solution. After a plateau was attained, cumulative doses (0.01–10 mg/ml) of RJ were added to obtain the concentration–response curves.

#### 2.4.2 Role of endothelium in RJ‐induced relaxation

To determine which endothelial mediators are associated with the vasodilation effects of RJ, the endothelium‐intact aortic rings were preincubated with 1 μM atropine (M_3_ receptor blocker), 100 μM L‐NAME (nitric oxide synthase inhibitor), 10 μM methylene blue (guanylate cyclase inhibitor), and 10 μM indomethacin (cyclooxygenase inhibitor) for 20 min before induction of a steady contraction by NE (1 μM), and then, RJ (0.01–10 mg/ml) was added cumulatively.

#### 2.4.3 Influence of Ca^2+^ channels on the effects of RJ

To illustrate whether the inhibition of extracellular Ca^2+^ influx or intracellular Ca^2+^ release has anything to do with the effect of RJ, the endothelium‐denuded aortic rings were incubated with K‐H solution containing 1 μM NE and then washed with Ca^2+^‐free K‐H solution until the contraction tension restored. After being incubated without or with RJ (0.03, 0.1 or 0.3 mg/ml) for 20 min, 1 μM NE was added to stimulate the release of intracellular Ca^2+^. When the tension was up to maximum, add 2.5 mM CaCl_2_ to induce the extracellular Ca^2+^ influx. In addition, the endothelium‐denuded aortic rings were washed with Ca^2+^‐free K‐H solution twice and then rinsed with Ca^2+^‐free K‐H solution (without EGTA) containing high KCl (60 mM). After 20‐min incubation without or with 0.3 mg/ml RJ, CaCl_2_ (0.5, 1, 1.5, 2, and 2.5 mM) was added cumulatively to obtain concentration–response curves, with the maximum contraction induced by the second administration of 60 mM KCl considered to represent 100%.

#### 2.4.4 Quantification of NO production and cGMP levels

The NO production and cGMP levels in the aortic rings were measured using NO colorimetric assay kit and cGMP ELISA kit of the Nanjing Institute of Bioengineering according to the manufacturer’s instructions. Briefly, 10% w/v aortic ring homogenates were made in prechilled saline using an IKA ULTRA‐TURRAX homogenizer (German). The obtained homogenate was centrifuged at 2,095 *g* for 20 min at 4°C, and the supernatants were stored at −80°C until used. The protein concentration of all samples was determined by Coomassie Brilliant Blue method, and then, NO production and cGMP levels were normalized to the total protein content of the samples.

### 2.5 Statistical analysis

Data were expressed as mean ± *SEM*. Statistical analysis was performed by one‐way ANOVA with Tukey’s multiple comparison test (Prism version 6.0, GraphPad Software, San Diego, CA). The maximal relaxant effect (*E*_max_) corresponds to the maximal amplitude of the highest concentration. The EC_50_ was defined as the concentration of RJ that induces 50% of maximal effect. *p* < 0.05 was considered significant.

## 3 RESULTS

### 3.1 Effect of RJ on SBP, DBP, and heart rate in SHR rats

**Figure 2:**
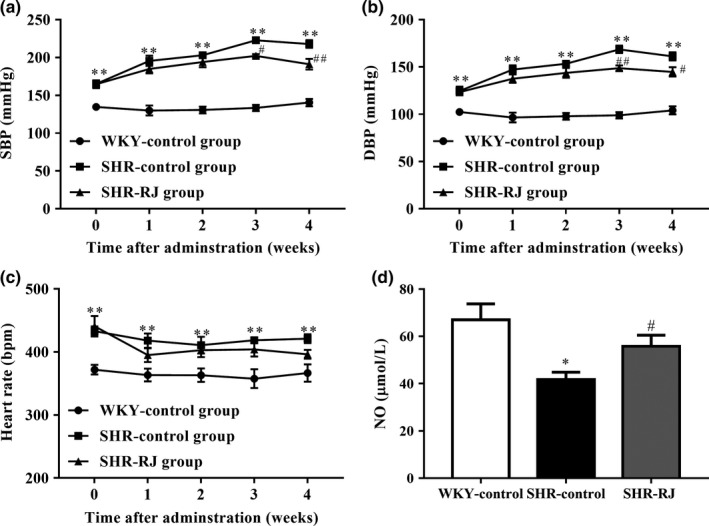
Effects of royal jelly’s (RJ) on systolic blood pressure (SBP), diastolic blood pressure (DBP), and heart rate in spontaneously hypertensive rats (SHR) rats. systolic blood pressure (SBP) (a), DBP (b), and heart rate (c) were monitored every week as well as NO level (d). Data are expressed as mean ± *SEM* (*n* = 6). *p < 0.05, **p < 0.01 compared with WKY‐control group, ^#^ *p* < 0.05, ^##^ *p* < 0.01 compared with SHR‐control group

As shown in Figure 2, the SBP, DBP, and heart rate of SHR were higher than those of WKY during the experimental period (*p* < 0.01). The SBP and DBP of SHR decreased from the third week of RJ treatment (*p* < 0.05, *p* < 0.01) compared with the SHR‐control group. But the heart rate has no differences between SHR‐RJ group and SHR‐control group (*p* > 0.05). Besides, RJ also increased NO level in SHR rats significantly (*p* < 0.05).

### 3.2 Vasorelaxant effects of RJ on NE‐primed aortic rings

**Figure 3:**
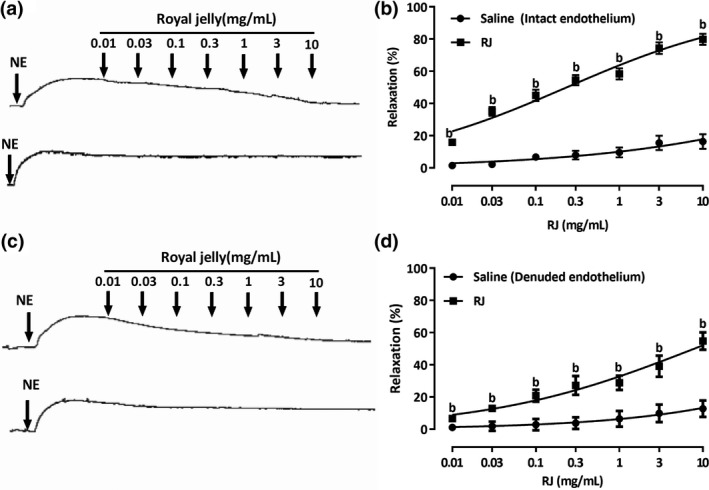
Vasorelaxant effects of royal jelly’s (RJ) on endothelium‐intact or endothelium‐denuded rings contracted with NE (1 µM). RJ dose‐dependently relaxed NE (a and b)‐contracted intact aorta or denuded aorta (c and d). The relaxant effects of RJ on isolated aortic rings were calculated as a percentage of the contraction in response to NE (1 µM). Data are expressed as mean ± *SEM* (*n* = 7–8). ^b^ *p* < 0.01 compared with the saline group

As shown in Figure 3 and Table 1, cumulative concentrations of RJ were applied into the tissue bath with 0.01–10 mg/ml every 10 min. The vasodilation effects of RJ in NE‐primed endothelium‐intact rings were achieved at EC_50_ and *E*_max_ value of 0.23 ± 1.17 mg/ml and 79.86 ± 3.42%, respectively. But the vasodilation of RJ was attenuated in NE precontracted endothelium‐denuded rings. The EC_50_ and *E*_max_ value of RJ were achieved at 7.88 ± 1.44 mg/ml and 54.81 ± 5.41%, respectively.

**Table 1:** Values of EC_50_ and *E*_max_ response on royal jelly’s (RJ)‐induced vasodilation to a different antagonist

Aortic rings condition/antagonist	EC_50_ (mg/mL)	*E*_max_ (%)
NE‐induced vasoconstriction (with endothelium)	0.23 ± 1.17	79.86 ± 3.42
NE‐induced vasoconstriction (without endothelium)	7.88 ± 1.44	54.81 ± 5.41^a^
Atropine	2.98 ± 1.43	55.58 ± 7.05^a^
L‐NAME	3.62 ± 1.38	53.40 ± 7.22^a^
Methylene blue	11.27 ± 2.02	43.28 ± 7.18^a^
Indomethacin	22.84 ± 2.11	41.64 ± 6.89^a^

Note

Data are expressed as mean ± *SEM* (*n* = 6–8).

a *p* < 0.01 compared with NE‐induced vasoconstriction (with endothelium).

### 3.3 Role of endothelium in RJ‐induced relaxation

**Figure 4:**
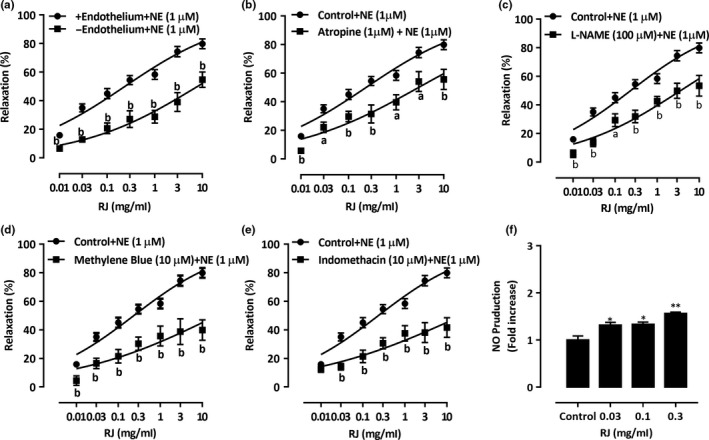
Role of endothelium in royal jelly’s (RJ)‐induced relaxation. Concentration‐dependent relaxant effect of RJ on NE (1 µM)‐contracted aortic rings with intact or denuded endothelium (a); Vasorelaxation of RJ was reduced by pretreatment of atropine (1 µM, b), L‐NAME (100 µM, c), methylene blue (10 µM, d), and Indomethacin (10 µM, e) in endothelium‐intact contraction induced by NE (1 µM). Data are expressed as mean ± *SEM* (*n* = 6–8). ^a^ *p* < 0.05, ^b^ *p* < 0.01 compared with control group. Endothelium‐intact rings were incubated with RJ (0.03–0.3 mg/ml), and levels of NO were determined (f). *n* = 3; *p < 0.05, ^**^ *p* < 0.01 compared to control

Removal of the endothelium had significantly attenuated the vasorelaxation effects of RJ (*p* < 0.01). About 25% of relaxation was abolished (Figure 4a), indicating the involvement of endothelium‐dependent vasorelaxing factors during vasodilation of RJ. Thus, the application of atropine had attenuated the RJ‐induced vasorelaxation in the endothelium‐intact rings, where EC_50_ value increased to 2.98 ± 1.43 mg/ml and *E*_max_ value of 55.58 ± 7.05% (*p* < 0.01, Figure 4b). Moreover, the vasodilation of RJ was markedly decreased in the presence of L‐NAME compared to the control, where EC_50_ and *E*_max_ values were achieved at 3.62 ± 1.38 mg/ml and 53.40 ± 7.22% (*p* < 0.01, Figure 4c), respectively. The presence of indomethacin has dramatically reduced the RJ‐induced vasorelaxation, where EC_50_ value increased to 22.84 ± 2.11 mg/ml, and *E*_max_ value to 41.64 ± 6.89% (*p* < 0.01, Figure 4e). Furthermore, the pretreatment of endothelium‐intact rings with methylene blue has decreased the RJ‐induced vasorelaxation, where EC_50_ and *E*_max_ values were achieved at 11.27 ± 2.02 mg/ml and 43.28 ± 7.18% (*p* < 0.01, Figure 4d), respectively. To further confirm the involvement of NO, rings were incubated with RJ (0.03–0.3 mg/ml) and then the level of NO was tested. As shown in Figure 4f, RJ caused a significant and dose‐dependent increase in NO production, which indicates that RJ exerts a vasorelaxant effect by increasing NO production.

### 3.4 Effect of RJ on the production of cGMP in aortic rings

**Figure 5:**
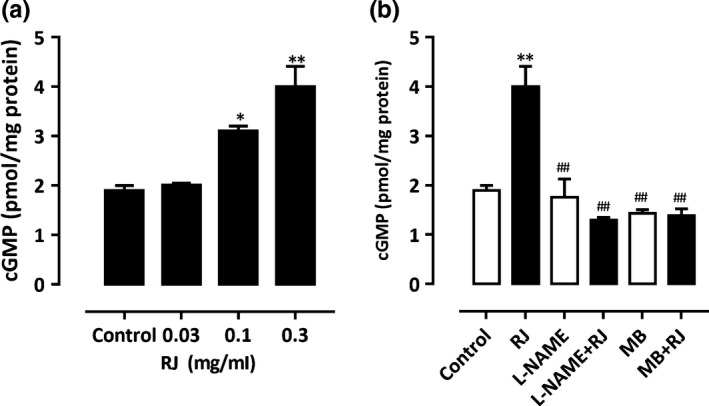
Modulation of cGMP levels by royal jelly’s (RJ), L‐NAME, and MB. (a) Rings were incubated in the absence (control) or presence of RJ (0.03–0.3 mg/ml). (b) Rings were pretreated with L‐NAME (100 µM) or MB (10 µM) for 20 min followed by RJ (0.3 mg/ml), and cGMP levels were determined. *n* = 3. *p < 0.05, ^**^ *p* < 0.01 compared to control; ^##^ *p* < 0.01 compared to RJ

Royal jelly’s‐induced vasorelaxation was significantly decreased by L‐NAME and MB, and these results suggested we have addressed the issue of whether RJ modulates the level of cGMP. Treatment with 0.03–0.3 mg/ml RJ evoked a significant and dose‐dependent increase of cGMP levels (Figure 5a). In addition, to confirm whether RJ‐increased cGMP accumulation is mediated by eNOS and/or sGC, rings were pretreated with either L‐NAME or MB followed by RJ treatment. The results showed that pretreatment with either inhibitor markedly inhibits RJ‐induced cGMP accumulation (Figure 5b).

### 3.5 Effect of RJ on Ca^2+^ channels

**Figure 6:**
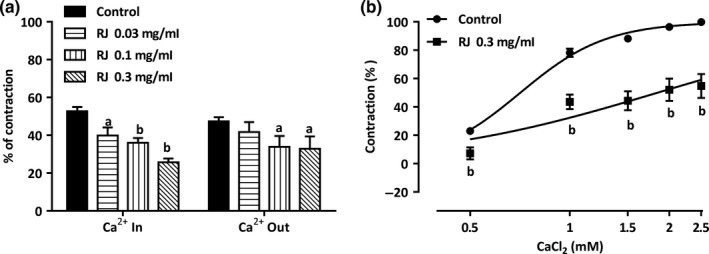
Effect of royal jelly’s (RJ) on Ca^2+^ channels in endothelium‐denuded aortic rings. (a) Vasodilation of RJ (0.03–0.3 mg/ml) is attributed to both intracellular calcium release and extracellular calcium influx. (b) RJ had an inhibitory effect on the cumulative‐contraction curve dependent on extracellular Ca^2+^ influx induced by KCl (60 mM) in Ca^2+^‐free solution. Data are expressed as mean ± *SEM* (*n* = 6–8). ^a^ *p* < 0.05, ^b^ *p* < 0.01

In the Ca^2+^‐free solution, pretreatment by RJ at 0.03–0.3 mg/ml significantly inhibited the contraction induced by NE (1 μM), *E*_max_ value decreased to 39.86 ± 4.30%, 36.01 ± 2.54%, and 25.66 ± 2.03%, respectively (vs. control group 52.71 ± 2.27%, Figure 6a), RJ could also inhibit the contraction induced by adding 2.5 mM CaCl_2_, and *E*_max_ value decreased to 41.71 ± 5.27%, 33.92 ± 5.72%, and 32.82 ± 6.54%, respectively (vs. control group 47.29 ± 2.27%, Figure 6a) in the endothelium‐denuded aortic rings. In addition, pretreatment with 0.3 mg/ml RJ significantly inhibited the contractions induced by extracellular CaCl_2_ (0.5–2.5 mM), with *E*_max_ value significantly reduced to 54.80 ± 8.43% (vs. control group 99.89 ± 0.11%, *p* < 0.01, Figure 6b) in the endothelium‐denuded aortic rings.

## 4 DISCUSSION

**Figure 7:**
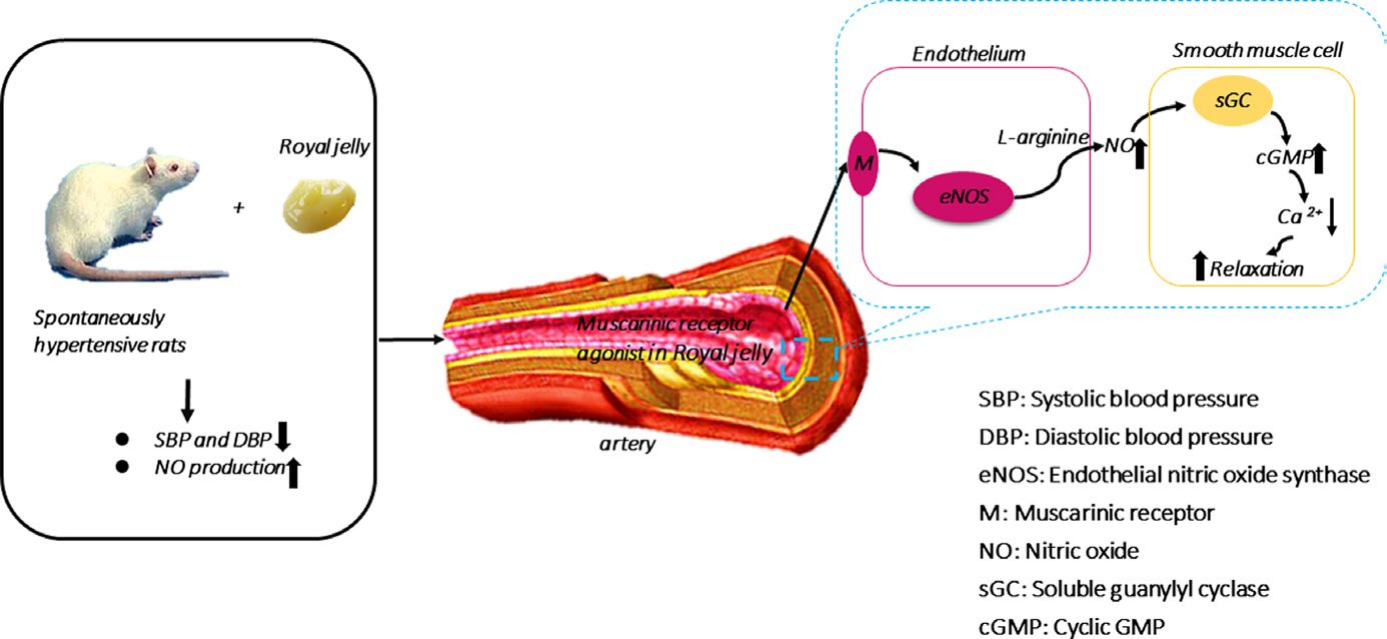
Schematic representation of royal jelly’s (RJ) lowers blood pressure and vasorelaxation by increasing nitric oxide production. RJ has antihypertensive effects and is associated with increased NO production. In addition, RJ contains muscarinic receptor agonists and induces vasorelaxation through NO/cGMP pathway and calcium channels

The present study confirmed that RJ has a hypotensive effect and can increase NO production in vivo. Furthermore, RJ contains muscarinic receptor agonists, likely ACh, and induces vasorelaxation through NO/cGMP pathway and calcium channels (Figure 7). In short, RJ induces hypotension and vasodilation by increasing NO production.

We then investigated whether the hypotensive effect of RJ is associated with an increase in NO production. To address this aim, the SHR model of hypertension, a gold standard for experimental studies of essential hypertension (Head, 1989; Tsuda & Masuyama, 1991), was used to study the antihypertensive effect of RJ. The cause of hypertension in SHR has been attributed to increased sympathetic adrenergic activity (Head, 1989). Arribas, Marin, Ponte, Balfagon, & Salaices (1994) found that β‐adrenergic receptor‐mediated aortic rings relaxation in SHR pretreated with NE was less than that in untreated SHR. This difference was linked to the impaired endothelial function. In the present study, oral administration of RJ decreased SBP and DBP as compared to SHR‐control groups, but had little effect on heart rate. Besides, a significant increase in NO was produced by the RJ‐treated SHR. Therefore, this study confirmed that oral administration of RJ can reduce blood pressure, and NO is responsible for causing arterial vasodilation in SHR rats induced by RJ.

We further investigated the vasodilation of RJ on isolated rabbit aorta rings. NE acts on the α receptor of the vascular smooth muscle cell (VSMC), activates receptor‐operated calcium channels (ROCCs) via the IP_3_ signaling pathway, and results in vasoconstriction (Janbaz et al., 2014; McFadzean & Gibson, 2002). RJ showed a vasorelaxant effect on NE precontracted aorta rings, and the effect was much stronger than which on the KCl models (the latter only relaxed 14%, data not shown), implying that the activation of K^+^ channels might not make a major contribution. Yet, the vasorelaxation response was not completely abolished by the endothelium‐denuded rings, which indicated that the vasodilator effects of RJ were mediated by endothelium‐independent pathway. Also, RJ significantly increased NO production in isolated aorta rings. Thus, endothelium‐dependent vasorelaxing factors may be involved in the vasodilation of RJ.

Of our interest, the presence of atropine, a nonselective antagonist that is normally activated by binding to acetylcholine and causing relaxation of VSMC (Yam, Tan, Ahmad, & Shibao, 2016), blocks RJ‐induced vasodilation. More importantly, the maximal effect of atropine on RJ (*E*_max_ value of 55.58 ± 7.05%) was comparable to that of endothelium‐denuded aorta rings (*E*_max_ value of 54.81 ± 5.41%). This suggests that muscarinic receptor agonist may be one of the vasodilators in RJ, such as ACh. As a matter of fact, it has been previously reported that RJ contains 912 μg/g of ACh‐like compounds (Wei, Min, Kang, Deng, & Lu, 2010). Besides, acetylcholine acts on muscarinic receptors on the vascular endothelial cells, and causing endothelial cells to release endothelium‐derived relaxing factors (EDRFs), such as nitric oxide (NO), prostacyclin (PGI_2_), and endothelium‐derived hyperpolarizing factor (EDHF). They are main factors that mediate endothelium‐dependent vasorelaxant effects (Bauer & Sotnikova, 2010; Lee et al., 2013; Sandoo et al., 2010). We also revealed that RJ‐induced vasodilation was attenuated by L‐NAME (nonselective eNOS inhibitor) and indomethacin (nonspecific COX inhibitor), further indicating that ACh‐like components of RJ also act on muscarinic receptor of vascular endothelial cells, and causing endothelial cells to release vasodilators NO and PGI2. NO is transformed by L‐arginine under the action of eNOS (Ameer et al., 2010), and it may pass through the endothelium into the vascular smooth muscle, activate the soluble guanylate cyclase (sGC), and promote relaxation in vascular smooth muscle by increasing the level of cyclic 3′,5′‐guanosine monophosphate (cGMP), which then stimulate protein kinase G (PKG) and result in vasodilation (Moncada, Palmer, & Higgs, 1991; Vaandrager & de Jonge, 1996). In the present study, MB (the cGMP‐dependent inhibitor) caused a dramatic reduction in the vasorelaxant effects of RJ and decreased cGMP levels. Thus, these results suggest that NO/cGMP cascade is involved in RJ‐mediated vasorelaxation effect. Additionally, the previous study has reported that a decrease in NO levels may cause a decrease in PGI2, and NO has a positive impact on PGI2 (Pelletier et al., 1998). Therefore, we believe that one of the vasodilation mechanisms of ACh‐like components in RJ is by increasing the production of NO in the vascular endothelium, which is consistent with a previous study (Shinoda et al., 1978). They also proved that RJ contains a vasodilation factor in the water‐soluble fraction and is considered to be ACh.

Acetylcholine is known to be rapidly degraded by acetylcholinesterase in the blood, which makes us wonder how much does muscarinic receptor agonist, such as ACh, in RJ needed to cause vasodilation in the blood. If multiple RJ administrations lead to the muscarinic receptor agonist accumulating in the blood, the heart rate may be directly reduced. However, we found that in addition to lowering blood pressure, RJ administration for 4 weeks did not significantly reduce SHR heart rate. Besides ACh being a useful biologically active compound in RJ, it also contains polyphenols, adenosine, and hormones such as progesterone, testosterone, and estradiol (Fratini, Cilia, Mancini, & Felicioli, 2016; Pasupuleti, Sammugam, Ramesh, & Gan, 2017; Ramadan & Al‐Ghamdi, 2012). On the one hand, ACh acts on the cardiac muscarinic receptor to slow down the heart rate, while adrenaline acts on the cardiac beta1 receptor to speed up the heart rate. These two effects are opposite, which may be one of the reasons for the insignificant change in heart rate. On the other hand, the decreased effect of blood pressure on stimulation of the sinus and the aortic arch baroreceptor is weakened, and the heart rate is accelerated by decompression reflex, which also are the causes of the inconspicuous decrease in heart rate. Certainly, the present study showed that atropine did not completely block the relaxation of RJ, suggesting that RJ may also contain other antihypertensive active ingredients. As reported, the antihypertensive active ingredient of RJ may be derived from the active polypeptide of its main protein‐1 which has been hydrolyzed by the gastrointestinal enzymes. These active peptides have an inhibitory effect on ACE activity and can reduce blood pressure by inhibiting the action of renin–angiotensin–aldosterone system. (Sultana et al., 2008). Previous studies have shown that under acidic conditions, the activity of the blood flow increasing factor (considered ACh) in RJ is relatively stable and shows good gastrointestinal stability (Shinoda et al., 1978). So far as we know, there are no data to prove that the ACh‐like component of RJ is identical to that of common ACh, and there are no reports that another component of RJ may have similar effects as muscarinic receptor agonists.

In addition, RJ is still able to relax the endothelium‐denuded aorta rings, indicating that RJ has a direct relaxation effect on VSMC. Although changes in the K^+^ channel activity of VSMC membranes regulate membrane potential, which is an important mechanism involved in arterial vasoconstriction and vasodilation (Kohler, Kaistha, & Wulff, 2010; Protic et al., 2015). However, RJ not only had weak vasodilation effect on the KCl models, and different types of potassium channel blockers also had no effect on RJ’s vasorelaxation (Data not shown). Therefore, RJ may not exert the function of vasorelaxation through the open K^+^ channels, but by inhibiting the intracellular Ca^2+^ pathway. Ca^2+^, derived from extracellular influx and intracellular release, is necessary for the contraction of smooth muscle (Ghosh et al., 2017). There are two major types of Ca^2+^ channels in the cell membrane, including receptor‐operated calcium channels (ROCCs) and voltage‐dependent calcium channels (VDCCs). NE is an α‐adrenergic receptor agonist that contracts smooth muscle cells not only through the extracellular Ca^2+^ influx in ROCCs but also through releasing intracellular Ca^2+^ from specific inositol 1,4,5‐triphosphate (IP_3_) receptor channel in sarcoplasmic reticulum (SR) membrane (Janbaz et al., 2014; Mcfadzean & Gibson, 2002). In Ca^2+^‐free K‐H solution, NE‐induced contraction is mainly mediated by the IP3 receptor (Karaki et al., 1997). In the present study, RJ inhibited NE‐induced contraction in the Ca^2+^‐free solution, indicating that the vasorelaxation effect of RJ may be related to intracellular Ca^2+^ released from specific IP3 receptor channels in the SR membrane. Besides, KCl is a membrane‐depolarizing agent that contracts smooth muscle cells mainly by the extracellular Ca^2+^ influx and subsequent opening of VDCCs (Nelson & Quayle, 1995). For example, pretreatment with high K^+^ could lead to the depolarization of the cell membrane and activation of VDCCs in Ca^2+^‐free K‐H solution, and adding CaCl_2_ would increase the extracellular Ca^2+^ influx followed by vasoconstriction (Peng, Xiong, & Wang, 2014). The results of this study also indicate that RJ can inhibit vasoconstriction induced by CaCl_2_. Thus, these results showed that the vasodilation effect of RJ was associated with calcium channels.

## 5 CONCLUSION

In this study, our results confirm that RJ has antihypertensive properties and is associated with increased production of NO. In addition, this study also found that RJ contains muscarinic receptor agonists, which induces vasorelaxation through NO/cGMP pathway and calcium channels. These findings support the hypothesis that by increasing NO production, RJ exerts the function of antihypertension and vasodilation.

## CONFLICT OF INTERESTS

The authors declare that they have no conflict of interests.

## ETHICAL STATEMENTS

All experimental protocols and procedures were reviewed and approved by the Institutional Animal Care and Use Committee of the Zhejiang Chinese Medical University (Protocol ZSLL‐2016–115).
